# Validation of the safety attitudes questionnaire (short form 2006) in Italian in hospitals in the northeast of Italy

**DOI:** 10.1186/s12913-015-0951-8

**Published:** 2015-07-24

**Authors:** Giang Nguyen, Nikoloz Gambashidze, Shoeb Ahmed Ilyas, Diana Pascu

**Affiliations:** Unit of Epidemiology and Medical Statistics, Department of Public Health and Community Medicine, University of Verona, Strada Le Grazie 8, Verona, 37134 Italy; New hospitals LTD, Tbilisi, Georgia; Ruby Med Plus, Jeddah, Saudi Arabia; Girolamo Fracastoro hospital, San Bonifacio, Verona, Italy

**Keywords:** Patient safety, Safety attitudes questionnaire, Italy, Questionnaire validation

## Abstract

**Background:**

Studying safety attitudes of front-line workers can help hospital managers take initiatives to improve patient safety. The Safety Attitudes Questionnaire, a psychometric tool that measures safety attitudes in health facilities, has been used and validated in several languages worldwide but there is no Italian version available. Hence, the study is aimed at cross-culturally validating the questionnaire (short form 2006) in Italian at two hospitals in the Veneto region (northeastern Italy).

**Methods:**

The translation and linguistic adaptation process of the questionnaire followed the World Health Organization guidelines. The questionnaire was delivered to staff working in four departments in two hospitals. Confirmatory factor analysis was used to assess the content validity of a pre-specified factor model that recognizes seven safety factors of the SAQ. Retest was performed to assess reliability. Internal consistency of items and safety factors was evaluated via Cronbach’s alpha.

**Results:**

Response rate was 60 % (*n* = 261/433). Test-retest correlation between items and factors showed a high degree of agreement. Goodness-of-fit indices demonstrated an acceptable hypothesis model with seven safety factors. Cronbach’s alpha of a whole questionnaire was 0.85, demonstrating a good internal consistency. Polychoric correlations showed that the factors are well correlated with each other. Stress recognition was found to have negative correlation with other safety factors.

**Conclusions:**

The Safety Attitudes Questionnaire in Italian language has satisfactory psychometric characteristics and is a valid instrument to measure safety culture in Italian hospitals.

**Electronic supplementary material:**

The online version of this article (doi:10.1186/s12913-015-0951-8) contains supplementary material, which is available to authorized users.

## Background

Healthcare, while considered safe, is a high-risk industry in which medical mistakes are likely to happen quite often. In European Union countries, the rate of adverse events that accompany healthcare recipients is around 8–12 % [1], and 50 % of them are preventable just by improving the culture for safety in hospitals [2].

Healthcare in Italy is provided by two types of trust: hospital trust and healthcare trust. Hospital trust is a tertiary care setting, which normally is teaching hospital. Healthcare trust, which is an integration of healthcare and social services, guarantees secondary acute care, primary and territorial care for residents in their territory. The national movement for patient safety started in 2004, with the publication of the report “*Risk Management in Healthcare: The problem of errors*” by the Ministry of Health. At the time, Italian hospitals were only at the beginning of their journey towards establishing patient safety as a basic, systematic and continuous care process. In January 2007, the Italian Ministry of Health established the National system for Patient safety, purporting to “*build and foster a patient safety culture*” [3] in the sense that hospital management boards and clinical risk managers need to monitor the safety performance, promote best practice, and have new methods to improve patient safety [4].

Surveying health workers using psychometric questionnaires is a way to investigate the existing safety culture in health facilities. To standardize this approach, a wide variety of psychometric questionnaires has been developed and published [5, 6]. These questionnaires often contain questions regarding several aspects of safety culture. Based on this information, healthcare managers could set up programs for their improvement [7].

The Safety Attitudes Questionnaire (SAQ) is a self-reported psychometric questionnaire developed to measure safety attitudes of front-line workers. In recent years, it has been the most commonly used questionnaire to measure safety culture [8]. The European Network for Patient Safety has recommended the SAQ as one of the three effective tools (alongside the Hospital Survey on Patient Safety Culture and the Manchester Patient Safety Assessment Framework) to be used in patient safety research [6]. It has been cross-culturally validated in different languages, including English [8], Norwegian [9], Turkish [10], Dutch [11], Chinese [12], Swedish [13], German [14], Portuguese [15], Arabic [16]. All these studies have showed that the SAQ possesses good psychometric properties in different languages. Moreover, when the questionnaire is externally validated, high scores of safety factors of the SAQ have demonstrated improvements of safety environment, such as lower rates of medical errors and inpatient mortality, shorter length of hospitalization [17, 18] or better working conditions, like lower rates of nurse turnover [8, 19].

So far, no evidence of the use of the SAQ in Italian language has been reported. Hence, this study is aimed at validating the Italian SAQ short form 2006 by evaluating its test–retest reliability, internal consistency, and the goodness-of-fit of the psychometric properties.

## Methods

### The questionnaire

The SAQ short form 2006 (available at https://med.uth.edu/chqs/surveys/safety-attitudes-and-safety-climate-questionnaire/) was translated independently into Italian by two native Italian translators. An Italian version with consensus on language was then issued through a reconciliation process and sent for back-translation. Following discussions between translators, an agreement was reached on the target language version; the back-translated version was then compared with the English one to make sure that the meanings were equivalent. A cognitive briefing was done with two risk-management nurses. The translation process followed World Health Organization (WHO) guidelines for linguistic validation of a questionnaire [20]. The Italian SAQ is found in additional file 1.

In the background information in the questionnaire, some participants’ qualifications were modified to reflect staff positions in Italian hospitals. The position “*Environmental support (housekeeper)*” was omitted because it was generally outsourced. “*Physician Assistant/Nurse Practitioner*” was translated as “*Medico/infermiere tirocinante*” which means doctor/nurse in training. “*Attending/Staff Physician*” and “*Fellow Physician*” were both translated as “*Medico Specializzando*”, a title given to physicians during their post-graduate specialization courses from the first to the last year.

### Questionnaire administration and participants

The study was carried out at teaching hospitals of the University of Verona and the Healthcare Trust 20 of Verona (so-called “G. Fracastoro hospital”) in Veneto region in Italy in April-May 2011. The participants were permanent staff working in Geriatrics, Surgery, Internal medicine and Obstetrics departments. The reason of selecting only permanent staff was to make the results comparable between the two hospitals, as at teaching hospitals there are healthcare professionals in training at all stages and in all specializations, while at G. Fracastoro hospital there are few, if none, medical professionals in training.

An anonymous questionnaire was delivered either during mid-day shifts or weekly meetings. Two weeks were given to participants to complete the questionnaire. Upon completion, participants returned the questionnaires in envelopes to the chief nurses. In case of non-response, a reminder was made by the chief nurses or the department directors, and another week was given to responders. In total, 433 questionnaires were sent out in the two hospitals.

The retest was performed two weeks after the test, in Geriatrics, Obstetrics and Internal medicine departments at G. Fracastoro hospital with the same procedure. The retest group was informed in advance. Of note, 57 questionnaires were sent out for retest purpose.

A covering letter in Italian language with clear statements on the aims of the study, information confidentiality, voluntary participation and purpose of the retest, was provided to respondents. The information was clearly explained to them by researchers. The study was approved by Head of the Unit of Hygiene and Preventive Medicine at the University of Verona, and Medical Boards of the hospitals.

### Hypothesized psychometric model of the questionnaire

The Italian SAQ contains 41 questions (or items) divided into seven safety attitudes factors which were: (i) Teamwork climate (perceived quality of collaboration among personnel), (ii) Safety climate (perceptions of a strong and proactive organizational commitment to safety), (iii) Job satisfaction (positive attitude about the work experience), (iv) Stress recognition (how stressors influence over performance), (v) Perceptions of hospital management, (vi) Perceptions of unit management and (vii) Working conditions (perceived quality of the work environment and logistical support). Items 2, 11 and 36 had reverse wording structure.

**Fig. 1:**
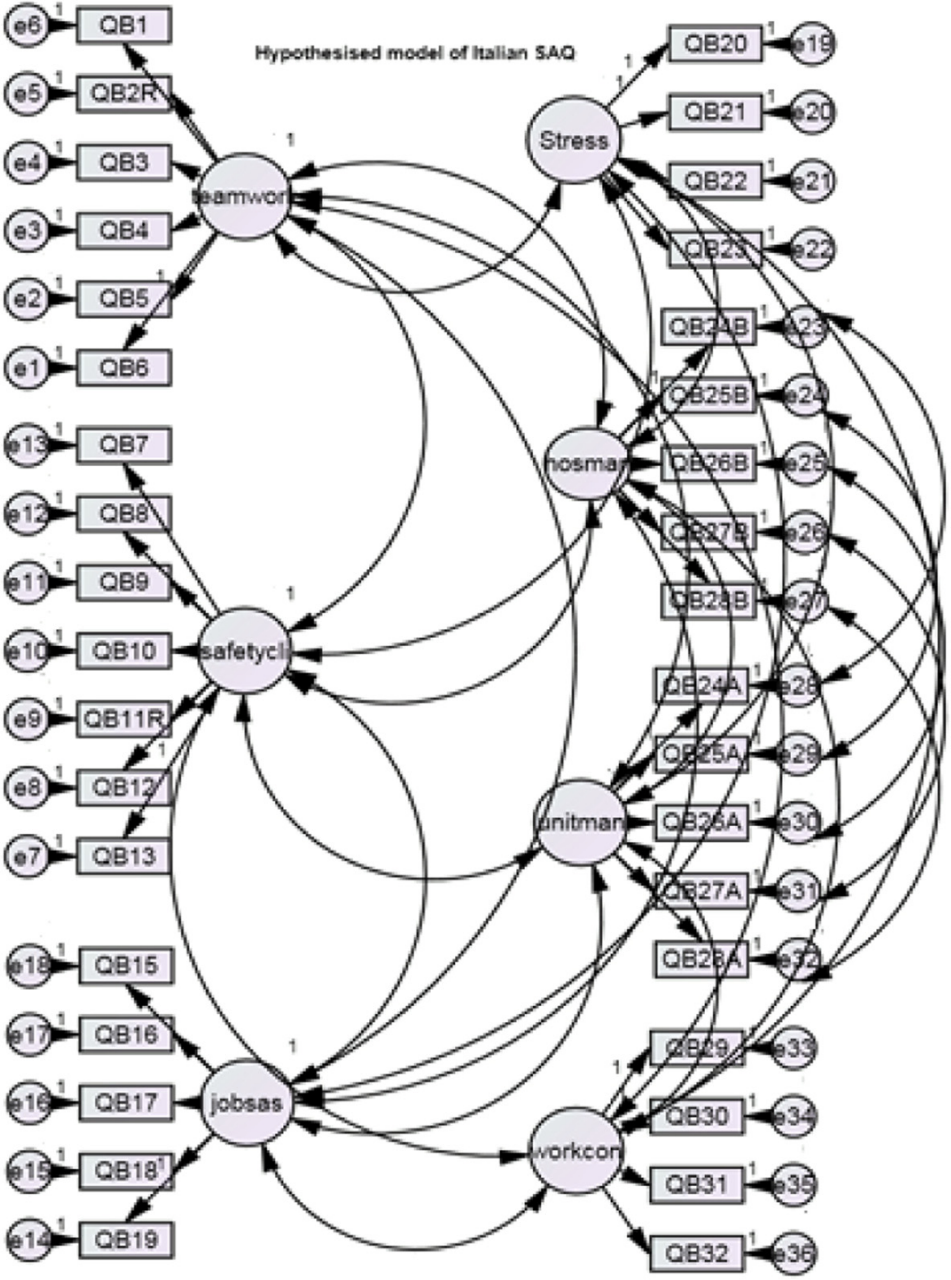
Hypothesized model of the Italian SAQ short form 2006

The hypothesized structural model was based on previous validation study in Norwegian language [9]. To the best of our knowledge, the structure suggested by the Norwegian team was the first validated structure of the SAQ short form 2006 ever published. This structure diverged to the one proposed by the Texas Center of Health Quality and Safety [21] on one specific point: the 29th item “*The levels of staffing in this clinical area are sufficient to handle the number of patients*” belongs to Working conditions factor instead of Perception of management. In fact, in the literature, this item was shown to be located in Working conditions in the SAQ ICU version [8], and in the Norwegian and Turkey SAQ short form version [9, 10]. Items 14, 33, 34, 35 and 36 did not belong to any safety factors. The model is described in Fig. 1.

### Data analyses

The SAQ used a 5-point Likert-scale (1 = *Disagree strongly*, 2 = *Disagree slightly*, 3 = *Neutral*, 4 = *Agree slightly* and 5 = *Agree*). Missing and “*Not Applicable*” answers were coded separately. The scale was treated as interval in the analysis [22]. Test-retest reliability was evaluated by Pearson’s correlation coefficient between items and factors at two points in time.

The psychometric hypothesized structure of the Italian SAQ was evaluated by Confirmatory Factor Analysis (CFA). In details, the following indices were considered: (i) the chi-square goodness-of-fit: the model is acceptable if the p-value of chi-square is not significant, (ii) relative chi-square, which is the chi-square divided by degree of freedom (d.f): the ratio ranges from 3 to 1 is acceptable for a model fit [23]; (iii) Comparative Fit Index (CFI) should lie within 0.90–1.00 for a fit model; and (iv) Root Mean Square Error of Approximation (RMSEA): a value of about 0.05 or less would indicate a close fit of the model in relation to d.f [24]. The Polychoric correlations between latent safety factors were calculated by the CFA.

The internal consistency was represented by Cronbach’s alpha (cut-off = 0.70) [25]. The value of Cronbach’s alpha is maximized when all items within one factor measure the same construct. Besides Cronbach’s alpha, item-rest correlation that displays correlation between one item and a scale computed from the rest items in the factor was also calculated for each item.

Once the structure was confirmed, the mean scores of safety factors of each respondent were calculated both in Likert-scale and 0–100 point scale to see the percentage of respondents having positive attitudes toward each safety dimension (≥ 75), equivalent to 4–5 points on the Likert-scale, using the formula:

*Mean score of a respondent = (mean of the factor items-1) × 25* [8].

A pair-wise criterion was used: if a respondent has more than two missing answers in a factor, their score is excluded from the score analysis for that factor [12]. The response options of reverse questions were converted into a positive scale before the mean of the factors was calculated. ANOVA test or Student’s *t*-test were performed to compare the mean scores of safety factors of different groups (hospitals, departments, working positions).

Analysis was performed in STATA InterCool 12.1 (Texas, TX, USA) and AMOS 19.0.0 (Meadville, PA, USA). P-value for significance is < 0.05.

## Results

### Characteristics of the respondents and response rates

Overall, the study response rate was 60.0 % (261/433) and retest response rate was 71.9 % (41/57). G. Fracastoro hospital recorded a high response rate of 71.1 % whereas that of teaching hospitals was 41.5 %. The majority of respondents were women (82.4 %) and nurses (51.3 %) (Table 1). Almost 60 % were staff working for more than 11 years in the field of medicine. The rates of missing answers ranged from 0 to 11 % with an average missing rate of 4.1 %. Items in Perceptions of unit management had highest missing rates (Additional file 2: Table S1). Percentage of positive attitudes of items separately varied from 17 % in the 24th item (*Hospital management supports my daily efforts*) to 90 % in the 15th item (*I like my job*) (Additional file 2: Table S1).

**Table 1:** Description of study respondents (n = 261)

Description	Number (%) (*n* = 261)	Description	Number (%) (*n* = 261)
Gender (female)	211 (82.4 %)	Answered SAQ before	
Hospitals		Yes	11 (4.2 %)
G. Fracastoro	168 (64.4 %)	No	238 (91.9 %)
Teaching hospitals	93 (35.6 %)	Don’t know	10 (3.9 %)
Department		Working experience	
Geriatrics	69 (26.4 %)	6 months	5 (2.0 %)
Obstetrics	48 (18.4 %)	6 to 11 months	10 (3.9 %)
Internal Medicine	57 (21.8 %)	1–2 years	22 (8.6 %)
Surgery	87 (33.4 %)	3–4 years	30 (11.8 %)
Position		5–10 years	36 (14.1 %)
Doctor	57 (21.8 %)	11–20 years	75 (29.4 %)
Chief nurse	6 (2.3 %)	More than 20 years	77 (30.2 %)
Nurse	134 (51.3 %)		
Clinical support	44 (16.9 %)		
Others	19 (7.3 %)		
Missing	1 (0.4 %)		

Time to complete the SAQ was from 10 to 15 min. However, physicians, more often than nurses, asked for clarification about reverse items and expressed their preference for those written in a normal sequence.

### Test and retest reliability

Pearson’s correlations of items between two times had a high degree of agreement, from good 0.46≤ k ≤0.74 (8 items), moderate 0.40≤ k ≤0.69 (17 items) (*p* < 0.05), to weak correlation for the rest of the items (Additional file 3: Table S2). Exceptionally, the coefficient of reversed item 11 was −0.11, showing little or no correlation between test and retest. Between safety factors, high Pearson’s correlation coefficients were recorded for Safety climate (*r* = 0.58), Job satisfaction (*r* = 0.83), Stress recognition (*r* = 0.61), Perceptions of hospital management (*r* = 0.80), Perceptions of unit management (*r* = 0.73); they were lower for Working conditions (*r* = 0.47) and Teamwork climate (*r* = 0.51). All the correlations were statistically significant (*p* < 0.05).

### Psychometric properties – construct validity and internal consistency

Goodness-of-fit indices for the hypothesized model and each factor separately are shown in Table 2. The questionnaire had significant chi-square test (*p* < 0.001) as a whole, but other indices were satisfied: a relative chi-square = 1.739, RMSEA = 0.05 and CFI = 0.90, lying within the acceptable range for a fit model. The total scale Cronbach’s alpha of the SAQ was 0.85, showing a good internal consistency. Cronbach’s alpha coefficients of each safety factor were within 0.70 and 0.86 (Table 2). Item-rest correlation coefficients are presented in the supplement Additional file 2: Table S1. Highest Polychoric correlation was observed between Teamwork climate and Safety climate (*r* = 0.95). Stress recognition had negative correlations to the other factors (Table 3).

**Table 2:** Psychometric properties of the Italian SAQ

SAQ factors	Fitness indices					Cronbach’s alpha
	*χ*2/d.f	p-value	RMSEA	p_close_	CFI	
Teamwork Climate	2.741	<0.001	0.08	0.07	0.93	0.73
Safety Climate	5.659	<0.001	0.13	<0.001	0.77	0.72
Jof Satisfaction	2.631	0.015	0.80	0.13	0.98	0.83
Stress Recognition	15.935	<0.001	0.24	<0.001	0.87	0.78
Perceptions of Hosp. Man.	2.541	0.018	0.08	0.15	0.98	0.84
Perceptions of Unit Man.	2.747	0.011	0.08	0.11	0.98	0.86
Working Conditions	7.635	<0.001	0.2	0.001	0.89	0.70
Whole model	1.739	<0.001	0.05	0.16	0.90	0.85

**Table 3:** Polychoric inter-correlations between factors

Safety factors	(1)	(2)	(3)	(4)	(5)	(6)	(7)
Teamwork Climate (1)	1.00	0.95	0.87	−0.16*	0.63	0.79	0.76
Safety Climate (2)		1.00	0.81	−0.27*	0.64	0.78	0.79
Job Satisfaction (3)			1.00	−0.25*	0.62	0.70	0.64
Stress Recognition (4)				1.00	−0.30*	−0.31*	−0.31*
Perc.of Hosp man. (5)					1.00	0.83	0.81
Perc.of Unit man. (6)						1.00	0.77
Working Conditions (7)							1.00

Polychoric inter-correlations between safety factors calculated by Confirmatory factor analysis. All correlations are significant (*p* < 0.05), except (*)

(Perc of Hosp man.: Perception of hospital management; Perc. of Unit man.: Perception of unit management)

### Mean score of safety factors

Table 4 displays mean scores of each safety factor in Likert-scale and 100-point scale. Stress recognition had the highest mean score compared with other factors (mean ± standard deviation: 75.8 ± 22.7). Meanwhile, mean scores of Perceptions of hospital management (49.4 ± 24.0) and Perceptions of unit management (53.4 ± 24.5) were relatively low.

**Table 4:** Mean scores (± standard deviation) of safety factors on Likert-scale and 100-point scale of the study population and of different groups’ characteristics (hospitals, working positions and working departments) (Perc of Hosp man.: Perception of hospital management; Perc. of Unit man.: Perception of unit management)

Factors	Teamwork Climate	Safety Climate	Job Satisfaction	Stress Recognition	Perc. of Hosp man.	Perc. of Unit man.	Working Conditions
Mean Likert ± SD	3.7 ± 0.6	3.6 ± 0.6	3.8 ± 0.9	4.0 ± 0.9	3.0 ± 1.0	3.1 ± 1.0	3.1 ± 0.9
Mean (100 scale) ± SD	66.4 ± 16.3	65.1 ± 14.9	70.6 ± 22.1	75.8 ± 22.7	49.4 ± 24.0	53.4 ± 24.5	51.6 ± 23.7
Hospitals							
G. Fracastoro	63.7 ± 15.0	64.3 ± 15.8	69.7 ± 23.7	76.1 ± 23.4	45.9 ± 24.9	52.8 ± 26.4	50.6 ± 23.8
Teaching hospitals	65.9 ± 11.8	66.4 ± 13.0	72.4 ± 18.8	75.0 ± 21.6	50.0 ± 22.5	60.9 ± 24.8	53.2 ± 23.4
p-value	0.24	0.27	0.34	0.70	0.20	0.018	0.39
Position							
Physician	74.4 ± 11.0	73.4 ± 12.9	77.9 ± 20.4	70.1 ± 27.3	54.0 ± 28.0	70.8 ± 27.0	63.6 ± 26.0
Nurse	60.1 ± 13.0	61.0 ± 15.4	66.8 ± 22.6	76.9 ± 21.9	42.9 ± 22.4	49.3 ± 25.0	44.6 ± 20.4
Chief nurse	67.5 ± 13.5	67.5 ± 11.0	75.3 ± 21.0	76.7 ± 17.5	50.0 ± 22.6	54.0 ± 23.0	57.7 ± 24.3
Clinical support	62.1 ± 14.3	63.2 ± 12.3	67.8 ± 20.9	77.4 ± 21.6	48.6 ± 20.5	53.3 ± 20.9	52.6 ± 23.0
Others	67.5 ± 13.5	67.5 ± 11.0	75.3 ± 21.2	76.7 ± 17.5	49.9 ± 22.6	54.0 ± 23.0	57.8 ± 24.3
p-value	<0.0001	<0.0001	0.004	0.22	0.009	<0.0001	<0.0001
Department							
Geriatrics	61.2 ± 14.4	65.6 ± 15.4	66.7 ± 20.2	76.4 ± 22.0	46.0 ± 21.7	51.8 ± 25.1	47.9 ± 21.8
Obstetrics	69.1 ± 13.6	67.7 ± 14.0	73.7 ± 21.3	82.2 ± 19.3	45.0 ± 24.9	54.5 ± 25.9	52.1 ± 20.3
Internal medicine	63.4 ± 12.4	61.8 ± 14.2	68.7 ± 23.1	78.7 ± 21.6	43.9 ± 22.9	54.1 ± 23.2	44.8 ± 23.1
Surgery	65.5 ± 14.2	65.4 ± 15.3	73.3 ± 23.0	69.6 ± 24.6	52.1 ± 25.8	60.8 ± 28.4	58.6 ± 25.6
p-value	0.022	0.24	0.19	0.096	0.17	0.19	0.003

Between the two hospitals, there was no difference in the percentage of the positive answers in each safety factors, except for Perception of unit management. When comparing different working positions, mean scores among physicians, nurses, chief nurses, clinical supports and other positions were significantly different in all safety factors but not in Stress recognition. Physicians and chief nurses gave more percentage of positive answers, while nurses got comparatively lower scores than others working positions. The lowest mean scores observed in nurses belong to Perceptions of hospital management (42.9 ± 22.4), Perceptions of unit management (49.3 ± 25.0) and Working conditions (44.6 ± 20.4). The difference of mean scores among departments was not statistically significant in almost all the factors. The only difference was found in Working conditions, that surgery department got a higher mean score compare to others (58.6 ± 25.6).

## Discussions

The study aims at cross-culturally validating the Italian version of the SAQ in two hospitals in Italy. Based on literature search, this is the first-ever study to do that. Response rate was 60 %, which is relatively lower than the international benchmarking of response rate (68 %) [8], or that of other validation studies [9, 10]. Overall, the Italian SAQ has shown satisfactory data on validity, displays a good internal consistency and a moderate correlation of test-retest reliability. The findings are comparable with the results of international benchmarking data [8], and other relevant studies using the SAQ in different languages [9, 10]. In line with previous SAQ validation studies, our study has supported the validity of the SAQ in Italian in measuring patient safety in hospitals.

Construct validity of the Italian SAQ, based on the goodness-of-fit indices, was not absolute but acceptable (*p* < 0.001). The p-value of the whole model is critical because it tends to accept complex models with many parameters and it disregards effect of sample size. On the other hand, other goodness-of-fit indices supported the fitness of the model (relative chi-square = 1.739, RMSEA = 0.05, CFI = 0.90). Cronbach’s alpha coefficients of all factors and items were within 0.70–0.90, confirming a very good internal consistency of the Italian SAQ [25]. The items were well located inside the safety factors that they are supposed to measure.

Correlation between latent factors achieved good values in our model, proving that they were well correlated with each other. All the correlations (except for Stress recognition) were more than 0.60, exceeding the international benchmarking for within-area correlations. The highest correlation observed between Teamwork climate and Safety climate (0.95, *p* < 0.001) was consistent with the results of the study by Sexton et al. [8].

Physicians preferred the items in normal wording to the reverse one. In our study, the reverse items had lower factor loadings than others, and the correlation of test-retest was negative (item 11th). Reverse wording is a method to control the agreement of responses so that responders must read carefully the questions before providing the answers [26]. However, studies have found that they may cause an unfavorable effect on psychometric properties [27], and measurement problems for the questionnaire [26]. Meanwhile items in normal wording structure could have higher correlation in test-retest reliability and higher internal consistency [28]. In our opinion, in order to keep the generality of the SAQ in different languages, reversed items should be retained as they are in the English SAQ. A brief reminder to participants to read carefully before each and every item is suggested to avoid unexpected effects of reverse wording.

The high rate of missing answers in Perceptions of hospital management and Perceptions of unit Management (7–11 %) was similar to the results of other studies using the SAQ in European countries [29]. The item with lowest percentage of positive answer (item 24th) also belongs to this safety factor. The publication of the SAQ international benchmarking likewise had the lowest mean score in Perceptions of management among all the safety culture factors [8]. In general, the existing hierarchical structure, i.e. the top-down management model, in hospitals in Europe could prevent unit staff from speaking out the issues or discussing them with the management [30]. Moreover, another possible explanation is the vague role of hospital managers to unit staff when the managers always work distantly. Thus, a “code of silence” is created in which the front-line staff cannot comment on perception of hospital management, safety events are not properly reported and risks are underestimated. This, in turn, affects patient safety in hospitals.

Working positions contributed significantly to difference in safety attitudes in hospitals (except for Stress recognition) in the present study. Low mean scores of six dimensions belonged to nurses. In general, nurses who spend more time in contact with patients suffer more stress. Meanwhile physicians who work more independently are more recognized by their fellows, and chief nurses have their own leadership and empowerment in the wards. Different studies in different medical settings and countries also exhibit different results. For example, mean scores in Working conditions and Perceptions of management were found to be lower in nurse compared with the scores in physicians in ICUs [18]. On the contrary, in the ambulatory setting in the United States, Modak et al. [31] found that attitude scores in Teamwork climate, Safety climate, Job satisfaction and Working conditions were similar in all health providers [31]. And Etchegaray et al. [32] found no difference of the mean scores in Job satisfaction between nurses and physicians in ICUs [32].

Stress recognition is the only factor in the SAQ that is put in terms of self-behavior of the respondents. The highest mean score of Stress recognition shows evidence of the awareness of workers about the effect of stressors on their performance [33]. However, this factor has been disputed since stress is a weakening factor to safety practice, and it has negative correlation with other safety factors. In the present study, in line with other studies, Stress recognition has negative correlation with other factors, presenting that it goes against safety practice in hospitals. The measurement of stress in this case does not contribute positively towards safety climate as it is supposed to [34], and is even removed from the SAQ Chinese final version [12].

The study findings are limited by the size and representativeness of the sample. The sample size was limited due to time constraints of the study. A convenience sampling was used for this collaborative study in order to define target departments in participating hospitals. Several methods were implemented to obtain a high response rate at G. Fracastoro hospital (71.1 %); for example, the questionnaires were delivered during mid-day shifts or physicians weekly meetings. Meanwhile, at teaching hospitals there were no such possibilities, making their response rate relatively lower (41.5 %) and no information was available for non-responders. Finally, external validation was not covered in the frame of the study; therefore no clinical relevance with the SAQ results could be obtained. While the external validation requires more hospitals participating [9], studies in the future should examine it with larger sample sizes.

## Conclusions

The Italian short form SAQ, when being administrated in one tertiary care acute hospital and one secondary care hospital in Italy with different safety cultures and different patient safety pathways and stories, had a moderate correlation of the test-retest reliability, acceptable goodness-of-fit, high internal consistency within factors, and high correlation between factors. The study found similarities and differences with previous validation studies of the SAQ in the world, which constituted a good evidence of questionnaire validation. The fact that the hospitals did not differ in their answers respect to the SAQ dimensions, except for Perception of unit management, has confirmed that the Italian version of the SAQ is suitable to be used in diverse hospital settings and a valid tool for measuring safety culture in Italian hospitals. External validation study using the Italian SAQ should be performed when it is possible.

## Additional files

Additional file 1: The Safety Attitudes Questionnaire (short form 2006) in Italian (PDF 275 kb) Additional file 2: Table S1. SAQ items description. (DOCX 21 kb) Additional file 3: Table S2. Pearson correlation coefficient for test-retest reliability. (DOCX 18 kb)

## Abbreviations

SAQ: Safety attitudes questionnaire
CFA: Confirmatory factor analysis
d.f: Degree of freedom
RMSEA: Root Mean Square Error of Approximation
CFI: Comparative Fit Index
WHO: World Health Organization
ICU: Intensive care unit
