# Supplementary material for: Validation of the safety attitudes questionnaire (short form 2006) in Italian in hospitals in the northeast of Italy
Source: BMC Health Serv Res. 2015 Jul 24;15:284. doi: 10.1186/s12913-015-0951-8 (PMC4512154; doi:10.1186/s12913-015-0951-8)
Supplement: Additional file 2: Table S1. — SAQ items description. (DOCX 21 kb) [file 12913_2015_951_MOESM2_ESM.docx]

Additional table S1: SAQ items description

|  | Missing  (%) | Mean (SD) | Agree^a^  (%) | Disagree^b^  (% ) | Item-rest correlation | Cronbach's alpha |
| --- | --- | --- | --- | --- | --- | --- |
| Teamwork Climate |  |  |  |  |  |  |
| 1. Nurse input is well received in this clinical area | 2% | 3.8 (1.2) | 69 | 20 | 0.49 | 0.68 |
| 2. In this clinical area, it is difficult to speak up if I perceive a problem with patient care. | 2% | 3.3 (1.4) | 51 | 34 | 0.31 | 0.74 |
| 3. Disagreements in this clinical area are resolved appropriately (i.e., not *who* is right, but *what* is best for the patient). | 2% | 3.8 (1.2) | 69 | 17 | 0.56 | 0.67 |
| 4. I have the support I need from other personnel to care for patients. | 3% | 3.9 (1.2) | 66 | 15 | 0.48 | 0.69 |
| 5. It is easy for personnel here to ask questions when there is something that they do not understand. | 1% | 4.2 (1.0) | 77 | 9 | 0.47 | 0.69 |
| 6. The physicians and nurses here work together as a well-coordinated team. | 1% | 3.7 (1.3) | 63 | 23 | 0.49 | 0.68 |
| Safety Climate |  |  |  |  |  |  |
| 7. I would feel safe being treated here as a patient. | 3% | 3.9 (1.1) | 63 | 14 | 0.47 | 0.68 |
| 8. Medical errors are handled appropriately in this clinical area. | 9% | 3.6 (1.0) | 47 | 11 | 0.58 | 0.65 |
| 9. I know the proper channels to direct questions regarding patient safety in this clinical area. | 3% | 3.8 (1.0) | 63 | 10 | 0.36 | 0.70 |
| 10. I receive appropriate feedback about my performance. | 3% | 3.4 (1.2) | 48 | 23 | 0.56 | 0.65 |
| 11. In this clinical area, it is difficult to discuss errors. | 1% | 3.0 (1.3) | 40 | 38 | 0.27 | 0.73 |
| 12. I am encouraged by my colleagues to report any patient safety concerns I may have. | 2% | 3.9 (1.1) | 62 | 12 | 0.36 | 0.70 |
| 13. The culture in this clinical area makes it easy to learn from the errors of others. | 3% | 3.7 (1.1) | 54 | 14 | 0.42 | 0.69 |
| Job Satisfaction |  |  |  |  |  |  |
| 15. I like my job | 2% | 4.7 (0.8) | 90 | 3 | 0.30 | 0.86 |
| 16. Working here is like being part of a large family. | 4% | 3.6 (1.3) | 57 | 20 | 0.71 | 0.77 |
| 17. This is a good place to work. | 2% | 3.6 (1.2) | 62 | 20 | 0.76 | 0.75 |
| 18. I am proud to work in this clinical area. | 1% | 3.9 (1.1) | 67 | 11 | 0.73 | 0.76 |
| 19. Morale in this clinical area is high. | 2% | 3.4 (1.3) | 52 | 28 | 0.60 | 0.80 |
| Stress Recognition |  |  |  |  |  |  |
| 20. When my workload becomes excessive, my performance is impaired. | 1% | 4.1 (1.2) | 76 | 11 | 0.60 | 0.71 |
| 21. I am less effective at work when fatigued. | 1% | 4.2 (1.1) | 81 | 11 | 0.68 | 0.68 |
| 22. I am more likely to make errors in tense or hostile situations. | 0.4% | 4.3 (1.1) | 84 | 10 | 0.57 | 0.73 |
| 23. Fatigue impairs my performance during emergency situations (e.g. emergency resuscitation, seizure). | 8% | 3.5 (1.4) | 53 | 24 | 0.47 | 0.80 |
| Perceptions of Hospital Management |  |  |  |  |  |  |
| 24. Hospital management supports my daily efforts. | 9% | 2.4 (1.2) | 17 | 47 | 0.59 | 0.77 |
| 25. Hospital management doesn’t knowingly compromise patient safety. | 10% | 2.9 (1.2) | 43 | 18 | 0.68 | 0.74 |
| 26. Hospital management is doing a good job. | 8% | 2.9 (1.2) | 25 | 31 | 0.59 | 0.77 |
| 27. Problem personnel are dealt with constructively by our hospital management. | 11% | 3.7 (1.3) | 22 | 33 | 0.53 | 0.79 |
| 28. I get adequate, timely info about events that might affect my work from hospital management | 6% | 3.0 (1.3) | 29 | 33 | 0.57 | 0.78 |
| Perceptions of Unit Management |  |  |  |  |  |  |
| 24. Unit management supports my daily efforts. | 8% | 3.5 (1.2) | 34 | 37 | 0.56 | 0.82 |
| 25. Unit management doesn’t knowingly compromise patient safety. | 10% | 2.7 (1.2) | 49 | 16 | 0.54 | 0.83 |
| 26. Unit management is doing a good job. | 9% | 2.9 (1.4) | 38 | 25 | 0.69 | 0.79 |
| 27. Problem personnel are dealt with constructively by our unit management. | 10% | 3.2 (1.3) | 34 | 31 | 0.69 | 0.78 |
| 28. I get adequate, timely info about events that might affect my work from unit management | 7% | 3.3 (1.3) | 42 | 28 | 0.64 | 0.80 |
| Working conditions |  |  |  |  |  |  |
| 29. The levels of staffing in this clinical area are sufficient to handle the number of patients. | 2% | 2.2 (1.4) | 25 | 66 | 0.39 | 0.69 |
| 30. This hospital does a good job of training new personnel. | 2% | 3.0 (1.3) | 38 | 40 | 0.62 | 0.54 |
| 31. All the necessary information for diagnostic and therapeutic decisions is routinely available to me. | 3% | 3.4 (1.2) | 46 | 23 | 0.52 | 0.61 |
| 32. Trainees in my discipline are adequately supervised. | 3% | 3.7 (1.2) | 56 | 20 | 0.40 | 0.68 |
| Other questions |  |  |  |  |  |  |
| 14. My suggestions about safety would be acted upon if I expressed them to management. | 5% | 3.0 (1.1) | 29 | 31 |  |  |
| 33. I experience good collaboration with nurses in this clinical area. | 0% | 4.4 (0.8) | 87 | 5 |  |  |
| 34. I experience good collaboration with staff physicians in this clinical area. | 0.4% | 4.0 (1.0) | 72 | 10 |  |  |
| 35. I experience good collaboration with pharmacists in this clinical area. | 10% | 3.6 (1.1) | 44 | 13 |  |  |
| 36. Communication breakdowns that lead to delays in delivery of care are common. | 3% | 3.2 (1.3) | 43 | 32 |  |  |

(a) The percentage of "Agree slightly" and "Agree strongly" answers

(b) The percentage of "Disagree slightly" and "Disagree strongly" answers
