# Supplementary material for: Validation of the safety attitudes questionnaire (short form 2006) in Italian in hospitals in the northeast of Italy
Source: BMC Health Serv Res. 2015 Jul 24;15:284. doi: 10.1186/s12913-015-0951-8 (PMC4512154; doi:10.1186/s12913-015-0951-8)
Supplement: Additional file 3: Table S2. — Pearson correlation coefficient for test-retest reliability. (DOCX 18 kb) [file 12913_2015_951_MOESM3_ESM.docx]

Additional table S2: Pearson correlation coefficient for test-retest reliability

| **Safety Climate dimensions** | **Item correlation** | **Dimension correlation** |
| --- | --- | --- |
| **Teamwork Climate** |  | **0.51*** |
| Q1: Nurse input is well received in this clinical area | 0.53** |  |
| 2. In this clinical area, it is difficult to speak up if I perceive a problem with patient care. | 0.51** |  |
| 3. Disagreements in this clinical area are resolved appropriately (i.e., not *who* is right, but *what* is best for the patient). | 0.46** |  |
| 4. I have the support I need from other personnel to care for patients. | 0.34* |  |
| 5. It is easy for personnel here to ask questions when there is something that they do not understand. | 0.3 |  |
| 6. The physicians and nurses here work together as a well-coordinated team. | 0.45** |  |
| **Safety Climate** |  | **0.58 **** |
| 7. I would feel safe being treated here as a patient. | 0.56** |  |
| 8. Medical errors are handled appropriately in this clinical area. | 0.24 |  |
| 9. I know the proper channels to direct questions regarding patient safety in this clinical area. | 0.24 |  |
| 10. I receive appropriate feedback about my performance. | 0.65** |  |
| 11. In this clinical area, it is difficult to discuss errors. | -0.11 |  |
| 12. I am encouraged by my colleagues to report any patient safety concerns I may have. | 0.08 |  |
| 13. The culture in this clinical area makes it easy to learn from the errors of others. | 0.67** |  |
| **Job Satisfaction** |  | **0.83 **** |
| 15. I like my job | 0.53** |  |
| 16. Working here is like being part of a large family. | 0.49** |  |
| 17. This is a good place to work. | 0.69** |  |
| 18. I am proud to work in this clinical area. | 0.68** |  |
| 19. Morale in this clinical area is high. | 0.59** |  |
| **Recognition of stress** |  | **0.61**** |
| 20. When my workload becomes excessive, my performance is impaired. | 0.57** |  |
| 21. I am less effective at work when fatigued. | 0.58** |  |
| 22. I am more likely to make errors in tense or hostile situations. | 0.45** |  |
| 23. Fatigue impairs my performance during emergency situations (e.g. emergency resuscitation, seizure). | 0.41* |  |
| **Perceptions of Hospital Management** |  | **0.80**** |
| 24. Hospital management supports my daily efforts. | 0.44** |  |
| 25. Hospital management doesn’t knowingly compromise patient safety. | 0.38* |  |
| 26. Hospital management is doing a good job. | 0.55** |  |
| 27. Problem personnel are dealt with constructively by our hospital management. | 0.64** |  |
| 28. I get adequate, timely info about events that might affect my work from hospital management | 0.62** |  |
| **Perceptions of Unit Management** |  | **0.73**** |
| 24. Unit management supports my daily efforts. | 0.48** |  |
| 25. Unit management doesn’t knowingly compromise patient safety. | 0.76** |  |
| 26. Unit management is doing a good job. | 0.66** |  |
| 27. Problem personnel are dealt with constructively by our unit management. | 0.58** |  |
| 28. I get adequate, timely info about events that might affect my work from unit management | 0.37* |  |
| **Working conditions** |  | **0.47**** |
| 29. The levels of staffing in this clinical area are sufficient to handle the number of patients. | 0.16 |  |
| 30. This hospital does a good job of training new personnel. | 0.34* |  |
| 31. All the necessary information for diagnostic and therapeutic decisions is routinely available to me. | 0.32 |  |
| 32. Trainees in my discipline are adequately supervised. | 0.23 |  |
| **Other questions** |  |  |
| 14. My suggestions about safety would be acted upon if I expressed them to management. | 0.37* |  |
| 33. I experience good collaboration with nurses in this clinical area. | 0.21 |  |
| 34. I experience good collaboration with staff physicians in this clinical area. | 0.57** |  |
| 35. I experience good collaboration with pharmacists in this clinical area. | 0.35 |  |
| 36. Communication breakdowns that lead to delays in delivery of care are common. | 0.13 |  |

*(*: p<0.05; ** p<0.01)*
