# Supplementary material for: Validation of the safety attitudes questionnaire (short form 2006) in Italian in hospitals in the northeast of Italy
Source: BMC Health Serv Res. 2015 Jul 24;15:284. doi: 10.1186/s12913-015-0951-8 (PMC4512154; doi:10.1186/s12913-015-0951-8)
Supplement: Additional file 1: The Safety Attitudes Questionnaire (short form 2006) in Italian — (PDF 275 kb) [file 12913_2015_951_MOESM1_ESM.pdf]

Atteggiamenti nei confronti della sicurezza: la prospettiva degli operatori di questa unità operativa

Lavoro presso (indichi l'unità operativa o il servizio in cui lavora di solito): ____________ che fa parte del Dipartimento ____________ Compili questo questionario con riferimento alla sua esperienza in questa unità operativa.

Cancelli in modo chiaro qualsiasi risposta voglia cambiare — Risposta corretta: ● — Risposta non corretta: ✓ ✗ ⊖ ◉

La preghiamo di rispondere alle domande che seguono facendo riferimento all'unità operativa in cui opera.
Scelga le sue risposte usando la seguente scala:

A	B	C	D	E	X
Fortemente in disaccordo	Un po' in disaccordo	Neutrale	Un po' d'accordo	Fortemente d'accordo	Non pertinente

Non pertinente / **Fortemente d'accordo** / **Un po' d'accordo** / **Neutrale** / **Un po' in disaccordo** / **Fortemente in disaccordo**

1. I suggerimenti dell'infermiere vengono accolti positivamente in questa unità operativa.	Ⓐ Ⓑ Ⓒ Ⓓ Ⓔ Ⓧ
2. In questa unità operativa è difficile parlare apertamente se percepisco un problema nella gestione di un paziente.	Ⓐ Ⓑ Ⓒ Ⓓ Ⓔ Ⓧ
3. I disaccordi in questa unità operativa sono risolti in modo adeguato (ovvero, non *chi* ha ragione , ma *cosa* è meglio per il paziente).	Ⓐ Ⓑ Ⓒ Ⓓ Ⓔ Ⓧ
4. Ricevo il supporto di cui ho bisogno dalle altre figure che si occupano dell'assistenza ai pazienti.	Ⓐ Ⓑ Ⓒ Ⓓ Ⓔ Ⓧ
5. Qui è facile per il personale fare domande quando non capisce qualcosa.	Ⓐ Ⓑ Ⓒ Ⓓ Ⓔ Ⓧ
6. Qui I medici e le infermiere lavorano insieme come una squadra ben coordinata.	Ⓐ Ⓑ Ⓒ Ⓓ Ⓔ Ⓧ
7. Da paziente mi sentirei sicuro ad essere curato qui.	Ⓐ Ⓑ Ⓒ Ⓓ Ⓔ Ⓧ
8. Gli errori medici sono gestiti appropriatamente in questa unità operativa.	Ⓐ Ⓑ Ⓒ Ⓓ Ⓔ Ⓧ
9. In questa unità operativa, conosco i canali appropriati per indirizzare le domande relative alla sicurezza del paziente.	Ⓐ Ⓑ Ⓒ Ⓓ Ⓔ Ⓧ
10. Ricevo feedback appropriati riguardo la mia performance.	Ⓐ Ⓑ Ⓒ Ⓓ Ⓔ Ⓧ
11. In questa unità operativa è difficile discutere degli errori	Ⓐ Ⓑ Ⓒ Ⓓ Ⓔ Ⓧ
12. Vengo incoraggiato dai miei colleghi a riferire qualsiasi preoccupazione posso avere riguardo la sicurezza del paziente.	Ⓐ Ⓑ Ⓒ Ⓓ Ⓔ Ⓧ
13. La cultura in questa unità operativa rende facile imparare dagli errori degli altri.	Ⓐ Ⓑ Ⓒ Ⓓ Ⓔ Ⓧ
14. I miei suggerimenti sulla sicurezza sarebbero messi in atto se io li facessi presenti alla direzione.	Ⓐ Ⓑ Ⓒ Ⓓ Ⓔ Ⓧ
15. Mi piace il mio lavoro.	Ⓐ Ⓑ Ⓒ Ⓓ Ⓔ Ⓧ
16. Lavorare qui è come fare parte di una grande famiglia.	Ⓐ Ⓑ Ⓒ Ⓓ Ⓔ Ⓧ
17. Questo è un bell'ambiente dove lavorare.	Ⓐ Ⓑ Ⓒ Ⓓ Ⓔ Ⓧ
18. Sono orgoglioso di lavorare in questa unità operativa.	Ⓐ Ⓑ Ⓒ Ⓓ Ⓔ Ⓧ
19. Il morale in questa unità operativa è alto.	Ⓐ Ⓑ Ⓒ Ⓓ Ⓔ Ⓧ
20. Quando il mio carico di lavoro diviene eccessivo, la mia performance ne risulta compromessa.	Ⓐ Ⓑ Ⓒ Ⓓ Ⓔ Ⓧ
21. Sono meno efficiente al lavoro quando sono stanco.	Ⓐ Ⓑ Ⓒ Ⓓ Ⓔ Ⓧ
22. È più probabile che commetta errori in situazioni di ostilità o tensione.	Ⓐ Ⓑ Ⓒ Ⓓ Ⓔ Ⓧ
23. La stanchezza compromette la mia performance nelle situazioni di emergenza (per esempio, manovre di rianimazione di emergenza, crisi epilettiche).	Ⓐ Ⓑ Ⓒ Ⓓ Ⓔ Ⓧ
24. La direzione supporta i miei sforzi quotidiani: direzione di UO Ⓐ Ⓑ Ⓒ Ⓓ Ⓔ Ⓧ direzione medica	Ⓐ Ⓑ Ⓒ Ⓓ Ⓔ Ⓧ
25. La direzione non mette consapevolmente a rischio la sicurezza del paziente: direzione di UO Ⓐ Ⓑ Ⓒ Ⓓ Ⓔ Ⓧ direzione medica	Ⓐ Ⓑ Ⓒ Ⓓ Ⓔ Ⓧ
26. La direzione sta facendo un buon lavoro: direzione di UO Ⓐ Ⓑ Ⓒ Ⓓ Ⓔ Ⓧ direzione medica	Ⓐ Ⓑ Ⓒ Ⓓ Ⓔ Ⓧ
27. I problemi personali vengono gestiti in modo costruttivo dalla nostra: direzione di UO Ⓐ Ⓑ Ⓒ Ⓓ Ⓔ Ⓧ direzione medica	Ⓐ Ⓑ Ⓒ Ⓓ Ⓔ Ⓧ
28. Ricevo informazioni adeguate e tempestive sugli eventi che possono influire sul mio lavoro da parte della: direzione di UO Ⓐ Ⓑ Ⓒ Ⓓ Ⓔ Ⓧ direzione medica	Ⓐ Ⓑ Ⓒ Ⓓ Ⓔ Ⓧ
29. I livelli di assunzione del personale in questo servizio sono sufficienti a gestire il numero di pazienti.	Ⓐ Ⓑ Ⓒ Ⓓ Ⓔ Ⓧ
30. Questo ospedale fa un buon lavoro per quanto riguarda la formazione del personale neoassunto.	Ⓐ Ⓑ Ⓒ Ⓓ Ⓔ Ⓧ
31. Tutte le informazioni necessarie per le decisioni diagnostiche e terapeutiche sono sempre a mia disposizione.	Ⓐ Ⓑ Ⓒ Ⓓ Ⓔ Ⓧ
32. I tirocinanti nella mia disciplina vengono adeguatamente supervisionati.	Ⓐ Ⓑ Ⓒ Ⓓ Ⓔ Ⓧ
33. Ho un buon rapporto di collaborazione con il personale infermieristico in questa unità operativa.	Ⓐ Ⓑ Ⓒ Ⓓ Ⓔ Ⓧ
34. Ho un buon rapporto di collaborazione con il personale medico in questa unità operativa.	Ⓐ Ⓑ Ⓒ Ⓓ Ⓔ Ⓧ
35. Ho un buon rapporto di collaborazione con la farmacia ospedaliera	Ⓐ Ⓑ Ⓒ Ⓓ Ⓔ Ⓧ
36. Sono frequenti problemi di comunicazione che portano a ritardi nell'erogazione delle cure.	Ⓐ Ⓑ Ⓒ Ⓓ Ⓔ Ⓧ

PRECEDENTI INFORMAZIONI

Ha già compilato in passato questo questionario? ☐ Sì ☐ No ☐ Non so

Data di oggi (mese/anno):______________

Posizione (segni una sola risposta)

- ☐ Medico frequentatore
- ☐ Medico specializzando
- ☐ Medico strutturato
- ☐ Medico/infermiere tirocinante
- ☐ Coordinatore infermieristico
- ☐ Infermiere
- ☐ Farmacista
- ☐ Terapista della riabilitazione
- ☐ Assistente sociale
- ☐ Dietista/nutrizionista
- ☐ OSS, OTA
- ☐ Tecnico (strumentista, di laboratorio, di radiologia, della prevenzione e gli assistenti sanitari)
- ☐ Amministrativo
- ☐ Addetti alle pulizie
- ☐ Altro dirigente (ad.es. primario)
- ☐ Altro:__________________________

Sesso: ☐ Maschio ☐ Femmina

Si occupa principalmente di: ☐ Adulti ☐ Bambini ☐ Entrambi

Anni di esperienza: ☐ Meno di 6 mesi ☐ 6-11 mesi ☐ 1-2 anni ☐ 3-4 anni ☐ 5 -10 anni ☐ 11-20 anni ☐ 21 anni o più

Grazie per aver compilato il questionario! - La ringraziamo del tempo che ci ha dedicato.

PER FAVORE NON SCRIVA IN QUEST'AREA

Copyright © 2004 by The University of Texas at Austin
Safety Attitudes Questionnaire Italian translated version, date 14 March 2011 San Bonifacio Hospital
